# Integrated analysis of cell shape and movement in moving frame

**DOI:** 10.1242/bio.058512

**Published:** 2021-03-26

**Authors:** Yusri Dwi Heryanto, Chin-Yi Cheng, Yutaka Uchida, Kazushi Mimura, Masaru Ishii, Ryo Yamada

**Affiliations:** 1 Unit of Statistical Genetics, Center for Genomic Medicine, Graduate School of Medicine Kyoto University, Kyoto, 606-8507, Japan; 2 Department of Immunology and Cell Biology, Graduate School of Medicine and Frontier Biosciences, Osaka University, Osaka, 565-0871, Japan; 3 Department of Intelligent Systems, Graduate School of Information Sciences, Hiroshima City University, Hiroshima, 731-3194 Japan

**Keywords:** Cell shape, Cell movement, Moving frame, Spherical harmonics, Integrated analysis

## Abstract

The cell's movement and morphological change are two interrelated cellular processes. An integrated analysis is needed to explore the relationship between them. However, it has been challenging to investigate them as a whole. The cell's trajectory can be described by its speed, curvature, and torsion. On the other hand, the three-dimensional (3D) cell shape can be studied by using a shape descriptor such as spherical harmonic (SH) descriptor, which is an extension of a Fourier transform in 3D space. We propose a novel method using parallel-transport (PT) to integrate these shape-movement data by using moving frames as the 3D-shape coordinate system. This moving frame is purely determined by the velocity vector. On this moving frame, the movement change will influence the coordinate system for shape analysis. By analyzing the change of the SH coefficients over time in the moving frame, we can observe the relationship between shape and movement. We illustrate the application of our approach using simulated and real datasets in this paper.

## Abstract (precis)

**Summary:** We developed a novel approach to integrate the analysis of cell shape and movement using moving frame.

## INTRODUCTION

The cell's movement and morphological change are highly integrated. They share many biological mechanisms controlled by the cytoskeleton, cell membrane, membrane proteins, and extracellular matrix (Friedl and Wolf, 2009; Ridley, 2003). Almost all forms of the cell's active movement need forces that are generated from dynamic shape change (Lämmermann and Sixt, 2009). Moreover, the difference in the shapes and sizes of motile cells reflects their movement pattern (Keren et al., 2008; Lämmermann and Sixt, 2009).

The recent advances in 3D-cell imaging and tracking have provided us with an ability to collect cell movement and morphological data simultaneously (Nketia et al., 2017; Maška et al., 2014; Meijering et al., 2012; Wang et al., 2008). It significantly facilitated researchers to study the dynamic interplay between shape and movement of the cell. Yet, it also introduces new challenges. First, we need the ability to objectively quantify the cell's shape and movement. Without this quantification, we cannot measure any standardized form of measurement that allows statistical procedures and mathematical calculations. Second, we need a novel computational method to integrate these data. This method should quantify the change of shape and the dynamic of movement simultaneously.

A variety of movement quantification can be straightforwardly computed. Given the trajectories, we can measure the total trajectory length, the distance between start and end point, the speed, and the acceleration of the cell (Meijering et al., 2012). However, cell trajectories usually suffer from noise, which may bias the results of some analyses. The smoothing procedures such as Gaussian Process (GP) (Mchutchon and Rasmussen, 2011) are necessary to address this problem.

The shape quantification is not as simple as movement quantification. Cells are three-dimensional (3D) objects that have arbitrary spatial positions, directions, and scales in 3D space. These shapes may be variations of the same shape and should be recognized as the same one. Thus, the shape objects should be normalized and put in a common frame of reference to make shape representations invariant under these isometric transformations (i.e. translation, scale, and rotation). Normalization for translation is easy to deal by translating the object so that its center of mass is at the origin. The scale normalization can be done by scaling the object in such a way that the surface area or volume is equal to 1. On the other hand, normalization for rotation is hard and a subject of many studies. The most well-known approach is the principal component analysis (PCA) based approach (Shilane et al., 2004). Another approach is to transform each shape into a function and then calculate the rotation that minimizes the distance between the two functions (Makadia and Daniilidis, 2003; Makadia et al., 2004).

We propose a more natural way to align the rotation of a moving object such as a motile cell. The cells rarely have a clear axis of orientation. However, when moving, we can define the front face of the cell as the most forward part of the cell. A frame of reference where the object direction becomes one of its axis and moves with the object along the trajectory is called moving frame (Hanson and Ma, 1995). This moving frame is constructed primarily using the velocity vector. Therefore, by using a moving frame as the reference frame, a change in the movement pattern will also change the shape quantification. Hence, this approach can bridge the analysis of shape and movement.

After normalization, we need a shape descriptor, which is a compact numerical representation of the 3D object shape. Spherical harmonic (SH) descriptor is a widely used descriptor to study the 3D cell shape (Shen et al., 2009; Ducroz et al., 2012; Du et al., 2013; Medyukhina et al., 2020). It is a spherical analogue of the 1-dimensional Fourier series. It considers a surface as a function on the unit sphere, which can be represented as a set of unique coefficients. In shape-movement analysis, SH was used to analyze amoeboid cell motion (Ducroz et al., 2012; Du et al., 2013) and to perform shape classification of motile cells (Medyukhina et al., 2020).

In this paper, we developed a framework that generates, standardizes, and integrates the shape and movement data of the cells. This framework comprises trajectory smoothing using GP, translation and scale normalization, rotation normalization using moving frame, and spherical harmonic decomposition for shape analysis. To illustrate our approach, we use both of simulated and real datasets.

## RESULTS

### Simulated dataset

The first step was to create a smooth trajectory from the observed data. Then, we realigned the shape on each time point by using moving frames that were created from the smooth trajectory. We extracted the shape-movement features from these realigned shapes.

To validate these, we created a simulated dataset that consisted of 250 data points. From these 250 data points, we selected 26 data points as observed data. The smooth trajectory and the shape-movement features that were extracted from these observed data should reflect the trajectory and features from the complete original data.

### The validation of the trajectory smoothing

**Fig. 1.**
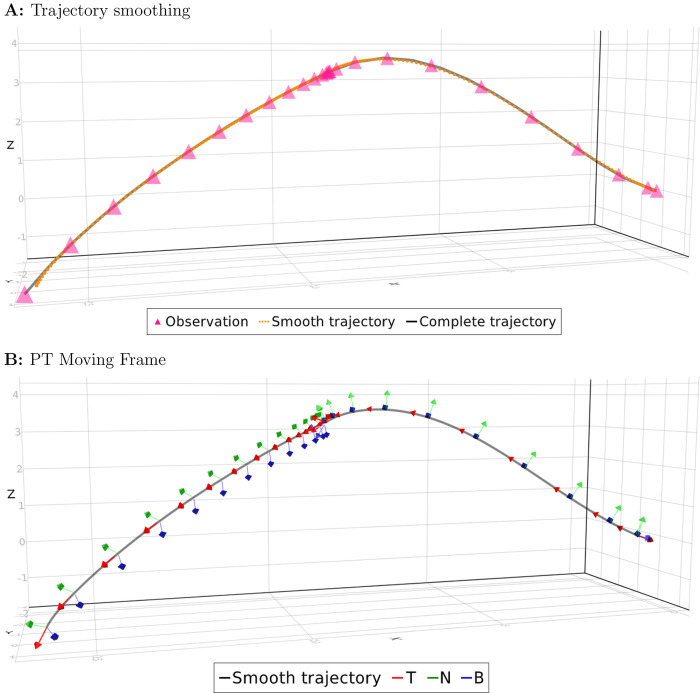
**Trajectory of simulated cell.** (A) The GP reconstructed the original trajectory from the observation. (B) The vector T together with the vectors N and B construct moving frames for each point on the curve.

We compared the smooth trajectory obtained from the observational data with the complete trajectory (Fig. 1A). It was difficult to see any difference due to almost perfect reconstruction (the mean squared error=1.203). The quality of the smooth trajectory degrades around *t*=0 and *t*=250. This is probably due to the fact that GP had fewer data to process around the boundary (i.e. no data at *t*<0 or t>250).

**Fig. 2.**
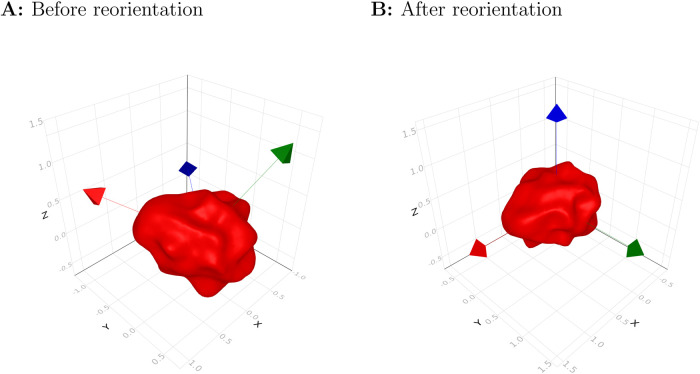
**Reorientation of shape using PT moving frame.** (A) Before the reorientation, we used the standard basis (e.g. X;Y;Z) to describe the shape of the 3D object. (B) After the reorientation, the moving frame becomes a canonical coordinate system to describe the shape. The moving frame consists of the tangent vector T (red), which is parallel to the direction of the movement, and two orthonormal vectors N and B (green and blue, respectively).

We constructed the parallel-transport (PT) moving frames on this smooth trajectory (Fig. 1B). Orientation normalization was performed using PT frames as the canonical frame of reference (Fig. 2). After reorientation, we observed the cell protruded its pseudopod toward the direction of movement (Movie 2b).

### Validation of the shape-movement features extracted from the observed data

**Fig. 3.**
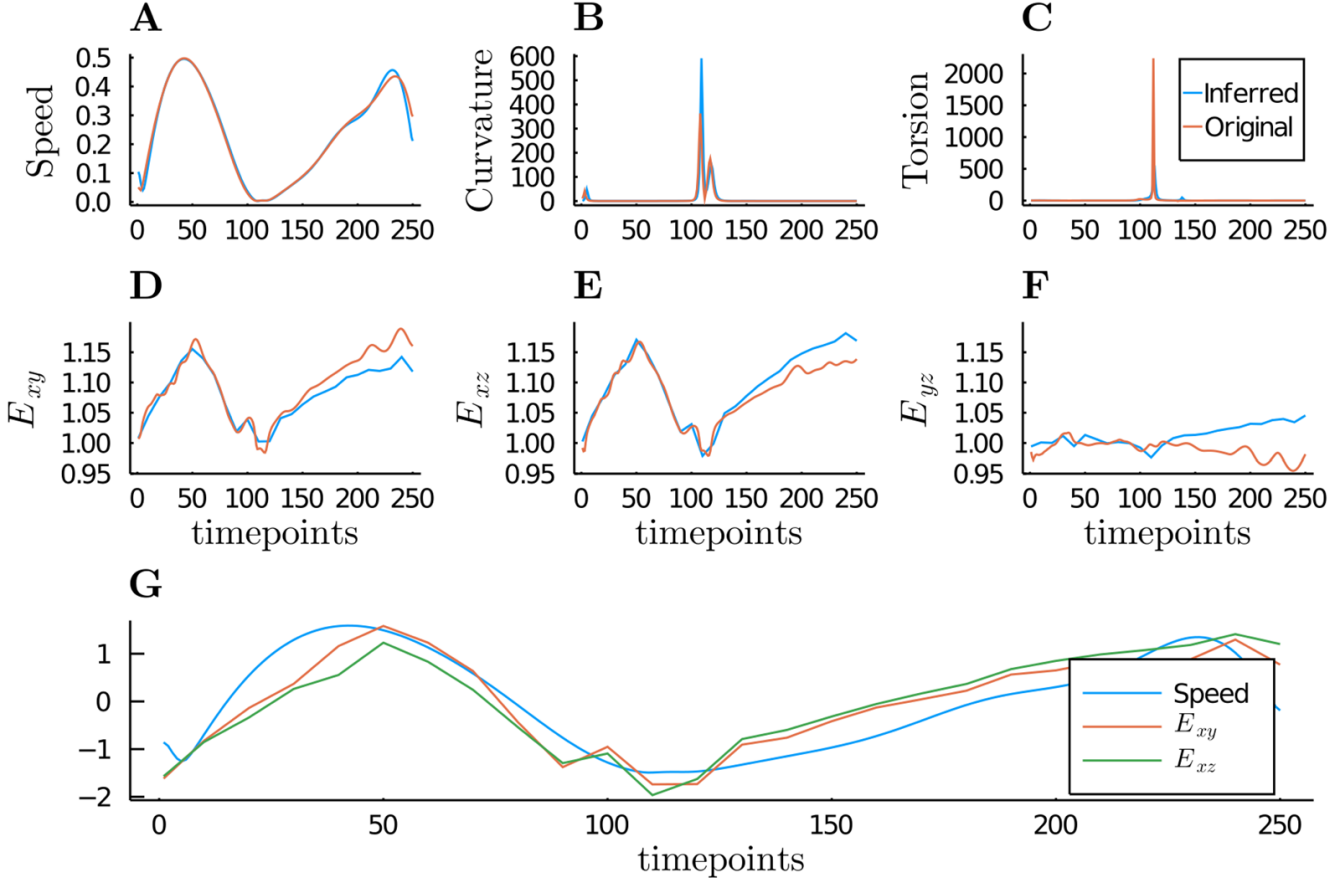
**The extracted shape and movement features from the smooth trajectory that is inferred from the observed data (label: inferred) and from the complete original data (label: original).** (A,B,C) The movement features of the cell are characterized by its speed, curvature, and torsion, respectively. (D,E,F) The global shape features of the cell is represented by the ratio of spherical harmonic coefficients. The patterns of the features that are extracted from the smooth trajectory are similar to the features from the complete data. (G) The relation between shape and movement of the cell can be seen in this graph. Here, we standardized the graph of speed, *E_xy_*, and *Exz* for easy comparison.

The shape-movement features of the simulated cell are shown in Fig. 3. The features from the complete original data are also shown on the graph. The pattern that we obtained from observational data was similar to the complete data, even though the magnitude of the peak was different. These differences are due to the data from observational data being smaller than the original data.

In the upper part of Fig. 3, the movement behavior of the cell is shown on the speed, curvature, and torsion graph. The bimodal graph of the speed graph indicates the accelerate-decelerate-accelerate-decelerate pattern. Meanwhile, the peak on the curvature and torsion graph indicates the change of the direction between *t*=100 and *t*=130.

In the middle part of Fig. 3, the global deformation patterns of the cell are shown as the eccentricity of the shape. Here, *E*_*xy*_ and *E*_*xz*_ changed as time progressed and had values more than 1 the majority of the time. In contrast, the value *E*_*yz*_ did not vary a lot from 1. These findings indicate that the cell had ellipsoid shape with the longer axis on the direction of the movement. Furthermore, the relation between shape and movement can be seen in the lower part of Fig. 3. In this plot, the speed, the *E*_*xy*_, and *E*_*xz*_ is standardized for comparison purpose. We can easily identify the similarity of the bimodal pattern on the speed, the *E*_*xy*_, and *E*_*xz*_ plot.

### Real data

For each cell in the real dataset, we aggregated the features from all time points and calculated its descriptive statistics measurements such as median, percentile, and median absolute deviation. These measurements can be readily used as the features for classification and visualization as shown in the latter paragraphs.

**Fig. 4.**
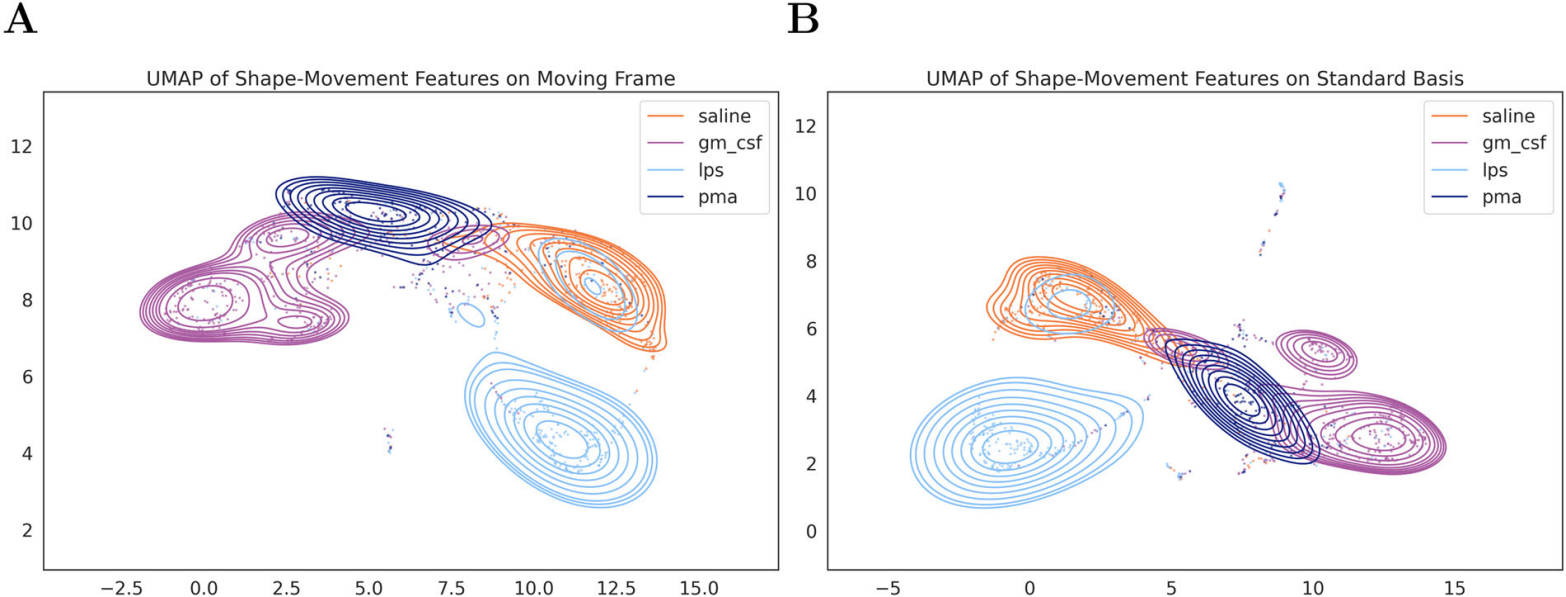
**The shape**-**movement features embedding on 2D space using UMAP.** We utilized UMAP to visualize the high-dimensional shape-movement features on (A) PT moving frame and on (B) standard basis.

To explore and visualize the high-dimensional shape-movement features, we used UMAP to plot the features of the real dataset on a two-dimensional (2D) plot. Fig. 4A and B show the 2D plot of the features from PT moving frame and the standard basis (i.e. no realignment). Qualitatively, the features obtained on the PT moving frame separate each group better than the features from the standard basis. From Fig. 4A, we can see that the saline group is separated from the treated groups [i.e. granulocyte-macrophage colony-stimulating factor (GM-CSF), lipopolysaccharide (LPS), and phorbol 12-myristate-13-acetate (PMA)]. Each of the GM-CSF, LPS, and PMA groups are also separated in different clusters.

**Fig. 5.**
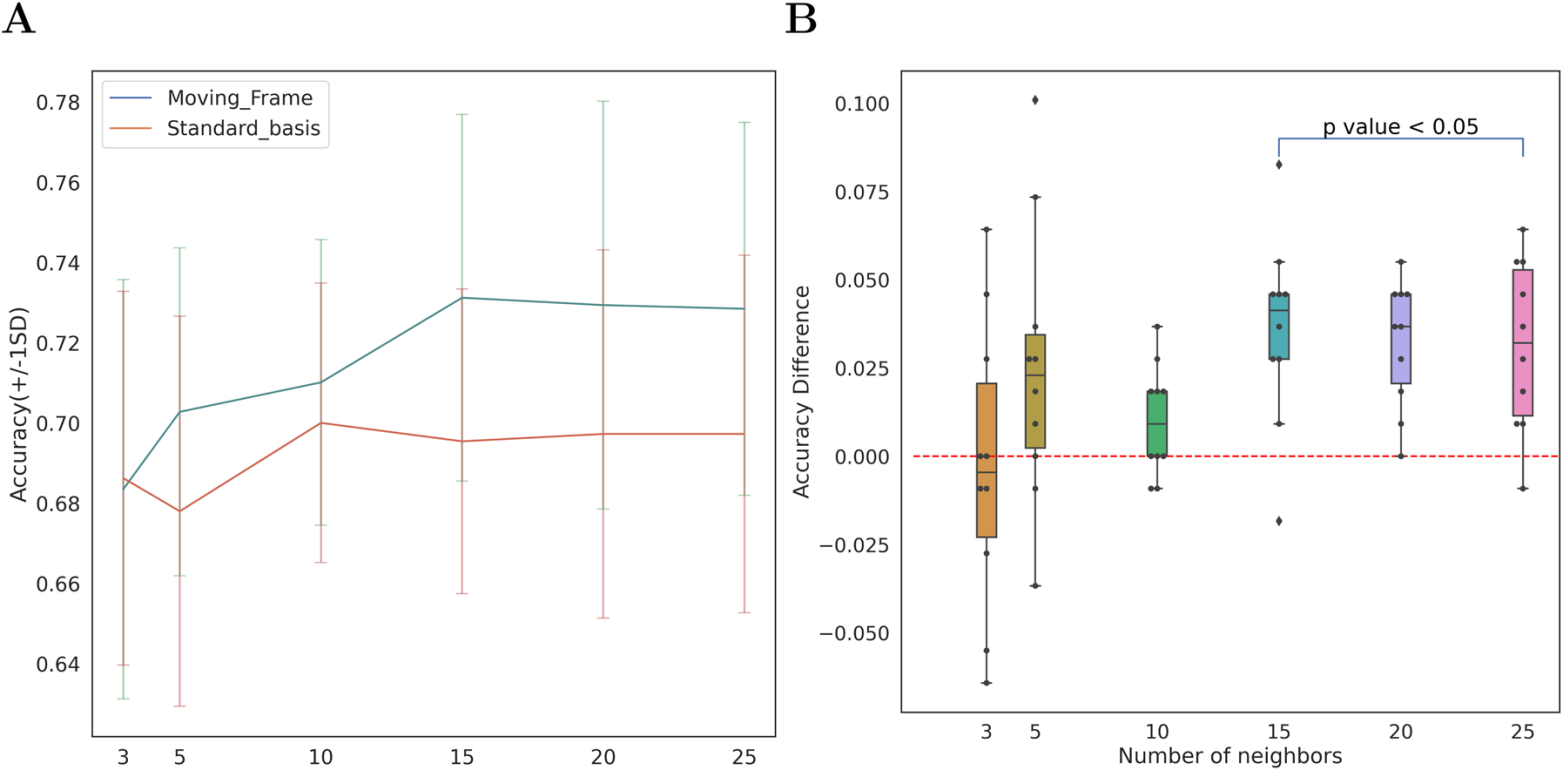
**The analysis of shape**-**movement features on PT moving frame.** (A) The comparison of accuracy of KNN classifier across tenfold cross-validation trained on PT moving frame and on standard basis. (B) The accuracy difference of KNN classifier on PT moving frame and on standard basis. The one-sample *t*-test showed that the difference was significantly different from 0 for *k*=15; 20; 25.

Quantitatively, we performed K-nearest neighbors (KNN) classifier on the features from the PT moving frame and the standard basis to show the performance of our approach. Fig. 5A shows the mean and the standard deviation of the KNN classifier accuracy. Most of the accuracy scores on the PT moving frame are superior to the standard basis. By using one-sample *t*-test, the accuracy difference is significant (i.e. *P*-value <0.05) on *k*=15,20,25 (Fig. 5B).

**Fig. 6.**
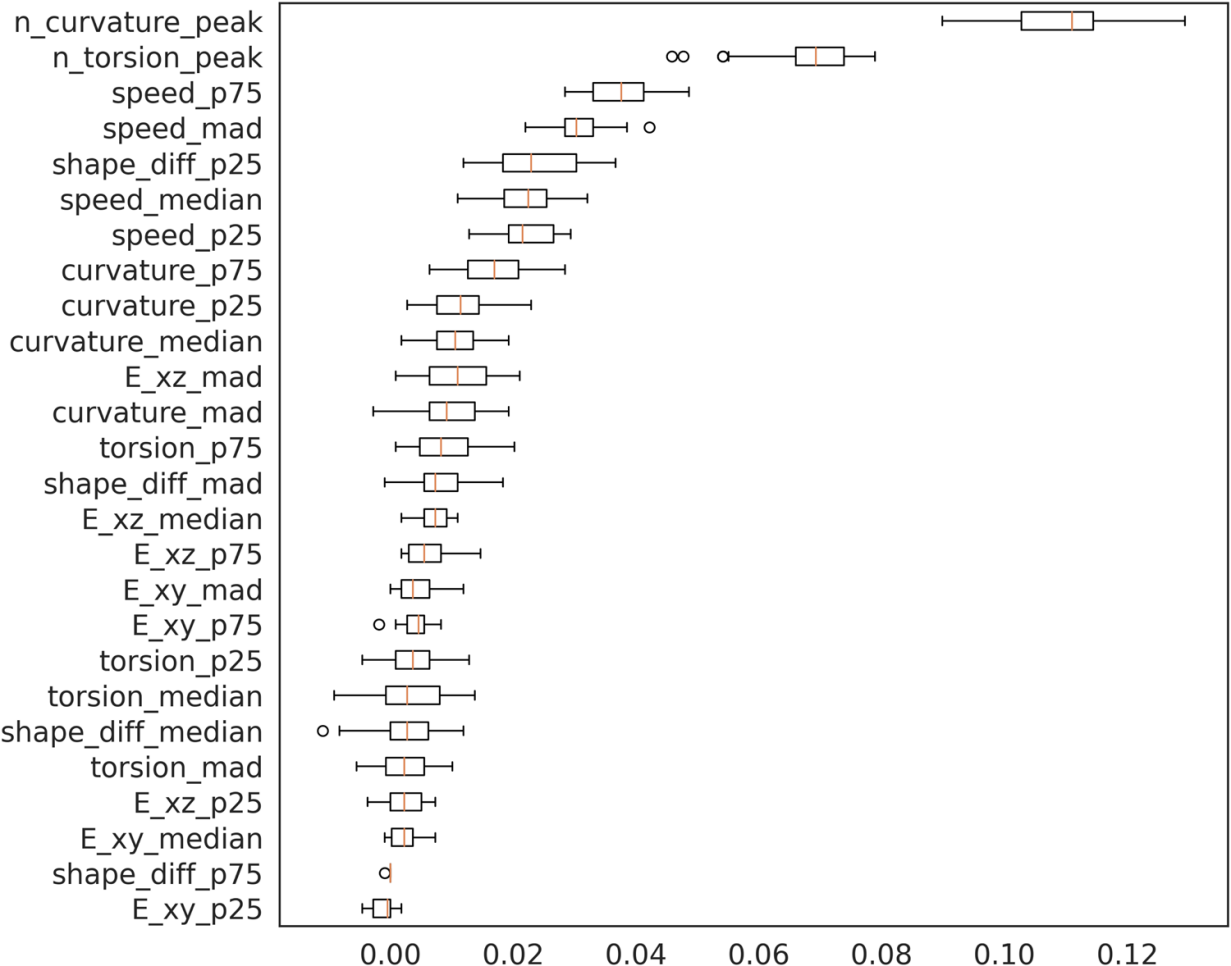
**The permutation importance plot shows the importance of each feature.** We found that movement features were dominantly selected as important features.

**Fig. 7.**
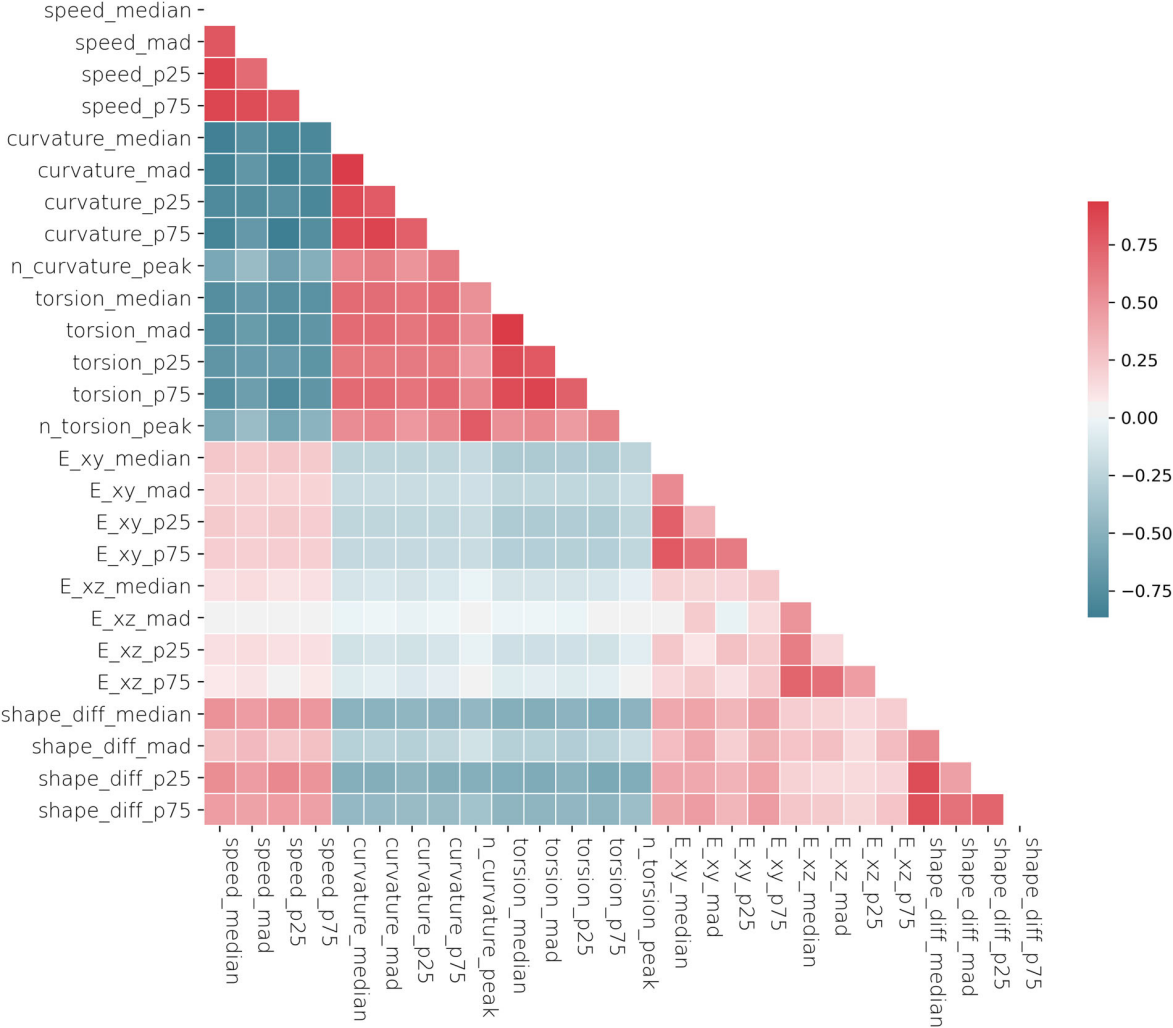
**The correlation matrix of each feature.**

In Fig. 6, we plotted the importance of each feature on the accuracy of KNN classifier. The most important features come from the speed features, which are the number of peaks on the curvature graph, the number of peaks on the torsion graph, the seventy-fifth percentile and median absolute deviation of the speed. Here, the shape features play a minor role to classify the sample. We can see the relationship between each feature in Fig. 7. We found some interesting observations from it; torsion and curvature features are negatively correlated with the speed features, which suggests that cells that are moving faster are inclined to move in a straight line (i.e. rarely change their direction and have a low magnitude of direction change). We also found that the fast cells’ shape was more ellipsoid and had a higher shape-change rate than the slower cells.

## DISCUSSION

In this proof-of-principle study, we developed a framework for an integrated analysis of the cell shape and movement data on moving frame. We illustrated the application of our framework on simulated and real data. Our approach can identify the relationship between shape-movement features such as the similarity between the speed and cell eccentricity pattern on the simulated dataset. Then, we can utilize the shape-movement features that are extracted by our framework for a further analysis or modelling. To illustrate this, we used shape-movement features extracted from our framework to visualize and classify neutrophils treated with some stimulants. We showed that our features could separate the groups of stimulants.

The key contribution of our approach is the capability to couple movement information with shape data using moving frame. The moving frame has many applications in the sciences such as in computer graphics (Hanson and Ma, 1995), medicine (Patil et al., 2015), and biology (Goriely et al., 2008). The moving frame has been applied to study the movement of organism and cells (Crenshaw, 1993; Crenshaw and Edelstein-Keshet, 1993). In this study, we used the moving frame as a canonical reference frame of the shape. It makes shape analysis task-independent to rotation transformation. Also, its construction is easy and fast. Other approachs, such as the PCA-based approach, rotation invariant shape descriptor, and estimation from spherical images, only use shape information to align the rotation (Kazhdan et al., 2003; Shilane et al., 2004; Makadia et al., 2004). We can integrate the shape-movement information because the moving frame is constructed using the velocity vector. In brief, when an object changes its movement (i.e. changes its velocity vector), the moving frame is simultaneously changed. The moving frame is the basis for shape descriptor. Thus, the change in moving frame will ultimately affect the shape analysis.

We need a smooth trajectory to construct moving frames. In our study, we used GP as a smoothing method. However, our approach is not restricted to this. We can also apply other smoothing procedures such as spline smoothing (Eubank, 1999), or the Savitzky-Golay filter (Savitzky and Golay, 1964). We chose GP because it can capture the uncertainty in the movements. These data can be useful for further movement analysis. GP is also more appropriate for our data because it does not need dense training data. The application of other smoothing methods should be studied in the future.

We used SH coefficient as a shape descriptor. The alternative descriptors are distribution-based (Osada et al., 2002), neural network-based (Fang et al., 2015), and wavelet-based descriptors (Laga et al., 2006). As an SH descriptor, they need shape normalization. Thus, our approach also applies for these descriptors. The performance of the descriptors should also be tested. The limitation of SH is the shape must be topologically spherical (i.e. there are no holes on it). A shape that is not topologically spherical cannot be mapped onto sphere using spherical parameterization. Despite this limitation, most of the cells analyzed were topologically spherical. We excluded cell that did not meet this requirement.

We used our approach to explore the shape-movement features of the neutrophil that were stimulated by GM-CSF, LPS, and PMA. We showed that our approach could extract and quantify the cell shape and movement information. The quantification allows us to perform statistical procedures, mathematical modelling, or classification. These reagents stimulate immune cells (Faurschou and Borregaard, 2003; Kutsuna et al., 2004; McAvoy et al., 2011). Thus, we expect our features can separate these groups from the saline control group. Indeed, we found that the treated groups are separated on UMAP visualization. Interestingly, each of the GM-CSF, LPS, and PMA groups had a different cluster. This indicates that each of the stimulants have different effects on cell shape and movement. This finding would be interesting to investigate further. For classification, we used KNN algorithm to measure how well the extracted shape-movement features captured the structure of shape-movement information in the dataset. Similar data points should close to each other. We showed that the shape-movement features that were obtained in moving frame were better than in the standard basis. Medyukhina et al. used the rotation-invariant SH classify migrating cells using Support Vector Machine (SVM) classifier (Medyukhina et al., 2020). However, the information about rotation is lost in the rotation-invariant SH (Kazhdan et al., 2003). Our approach preserved the rotation information (e.g. when the cell rotated, the moving frame also rotated).

We also measured the correlation between cell protrusion and movement. It is well known that one of the cell migration modes is by generating protrusion (reviewed in Yamada and Sixt, 2019). Yet, it is difficult to quantify the correlation. It is because of no standardized way to determine the cell front. By moving frame, we can solve this problem easily. Using our approach, we found that the fast cells have an ellipsoid shape with one of its principal axes angled towards the direction of movement. This ellipsoid shape might be because the cell is protruding but keeping the volume constant. It agrees with the experimental observation in Yamada and Sixt (2019).

Despite the advantages, our integrated analysis approach has limitations. This approach needs high resolution shape data. The low resolution could create an inaccurate trajectory due to imprecise calculation of the cell center. We also need many data points (e.g. preferably more than ten) to create a smooth trajectory.

Taken together, we showed that our framework can provide standardized measurement of cell shape-movement information and integrates these data with the appropriate resolution and number of data. This ability helps to gain insight into the mechanobiological process of the cell. However, some limitations may apply to this work.

## MATERIALS AND METHODS

### Overview

**Fig. 8.**
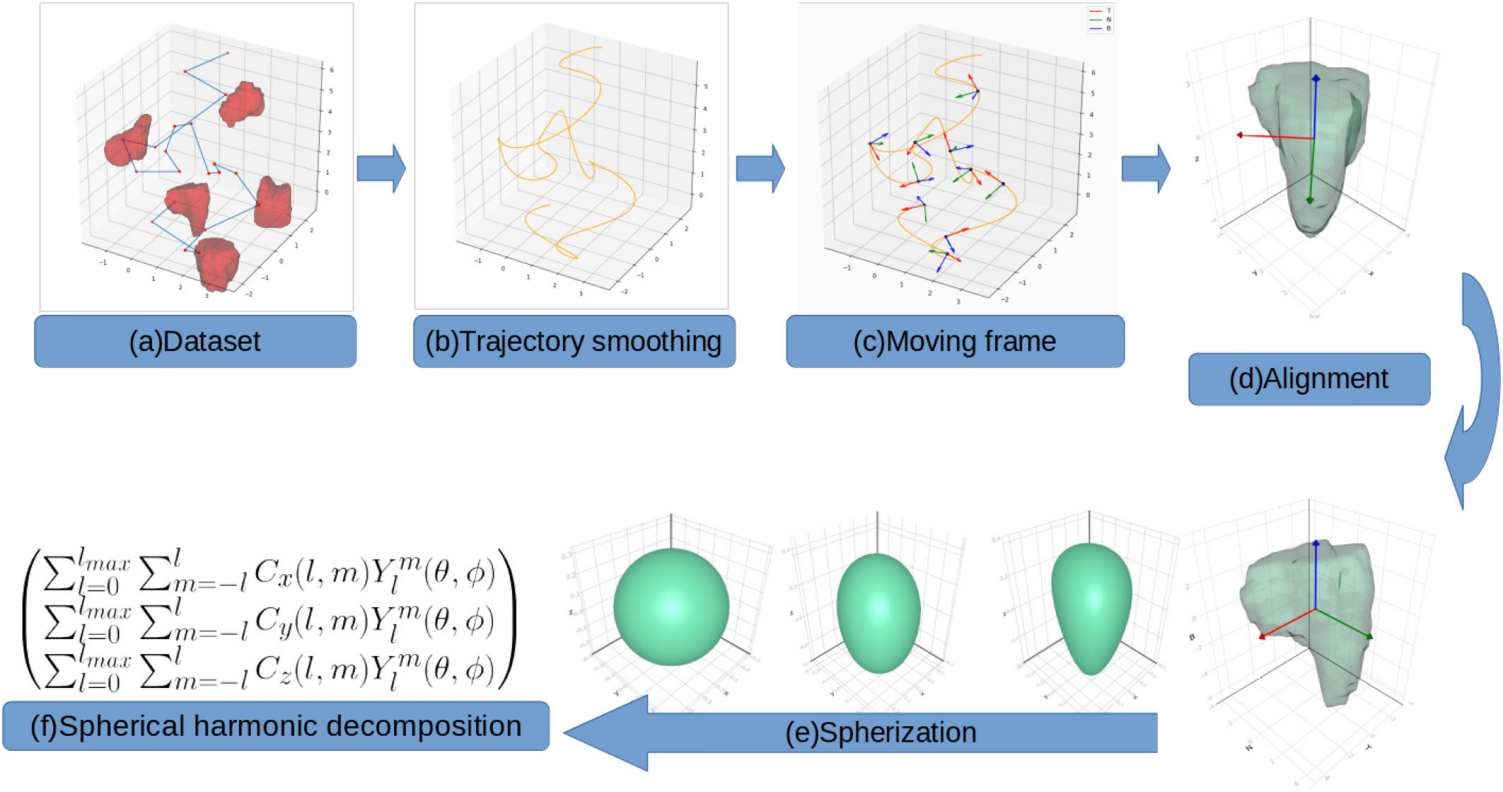
**Schematic diagram of integrated analysis of shape and movement in moving frame.**

The schematic diagram of our approach is shown in Fig. 8. The trajectory of the cell was smoothed using GP regression. From this smooth curve, we constructed moving frames at each sample point and extracted the trajectory properties such as the speed, curvature, and torsion at each sample point. For each 3D object from each time point we changed the coordinate basis from the standard basis to the moving frame basis. In this moving frame, the cell direction become the new x-axis. We performed the SH decomposition on this new basis to obtain SH coefficients. By analyzing the change of these coefficients over time, we can observe the dynamic of shape change when it was moving. We performed the statistical analysis of the shape and movement features to study their relationship.

### Trajectory and moving frame of the mass center

#### GP smoothing

We obtained the trajectory by calculating the mass center of the 3D object at every time point. The mass center of the object is defined as the arithmetic mean of their vertices. Thus, we had (*x*(*t*), *y*(*t*), *z*(*t*)) as the position of the mass center at the time *t*. Then, we defined *s*_*x*_=(*x*(1),..,*x*(*n*)), *s*_*y*_=(*y*(1),..,*y*(*n*)), and *s*_*z*_=(*z*(1),..,*z*(*n*)). For each sequence *s*_*i*_;*i*=*x*, *y*, *z*, we standardized the sequence so that it has mean zero and standard deviation one. From hereafter, the subscript *i* refers to one of the element of *x*, *y*, *z*, unless otherwise stated.

We modeled each of the sequence as 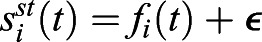 where 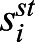 is a standardized sequence. The function *f*_*i*_(*t*) is an underlying smooth function and *ε* is a Gaussian noise with mean zero and variance *σ*^2^.

The function *f*_*i*_(*t*) can be approximated by GP regression (Rasmussen and Williams, 2006). In the GP regression, we have mean function and kernel function as hyperparameters. The mean function is a function that calculates the mean at any point of the input space and the kernel function specify the covariance between pairs of input. As our sequence had zero mean, we chose the zero mean function. And because we need a smooth function, we used Squared-Exponential (SE) kernel function.

The SE kernel itself has two parameters: (1) the length-scale parameter that determines how far the influence of one sample point to its neighbor points, and (2) the variance parameter that determines the mean distance from the function's mean.

We used the function *f*_*i*_(*t*) to interpolate new data points between two consecutive elements of sequence 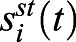 and 
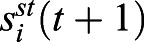
. The number of new data points determines the smoothness of the new sequence.

Next, we performed the inverse transformation of standardization by multiplying 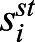 by the standard deviation of *s*_*i*_, then adding it together with the mean of *s*_*i*_ to produce a near-smooth sequence *r*_*i*_. Then, we defined *r*(*t*)=(*r*_*x*_(*t*), *r*_*y*_(*t*), *r*_*z*_(*t*)) as the smooth trajectory.

#### Parallel transport moving frame

**Fig. 9.**
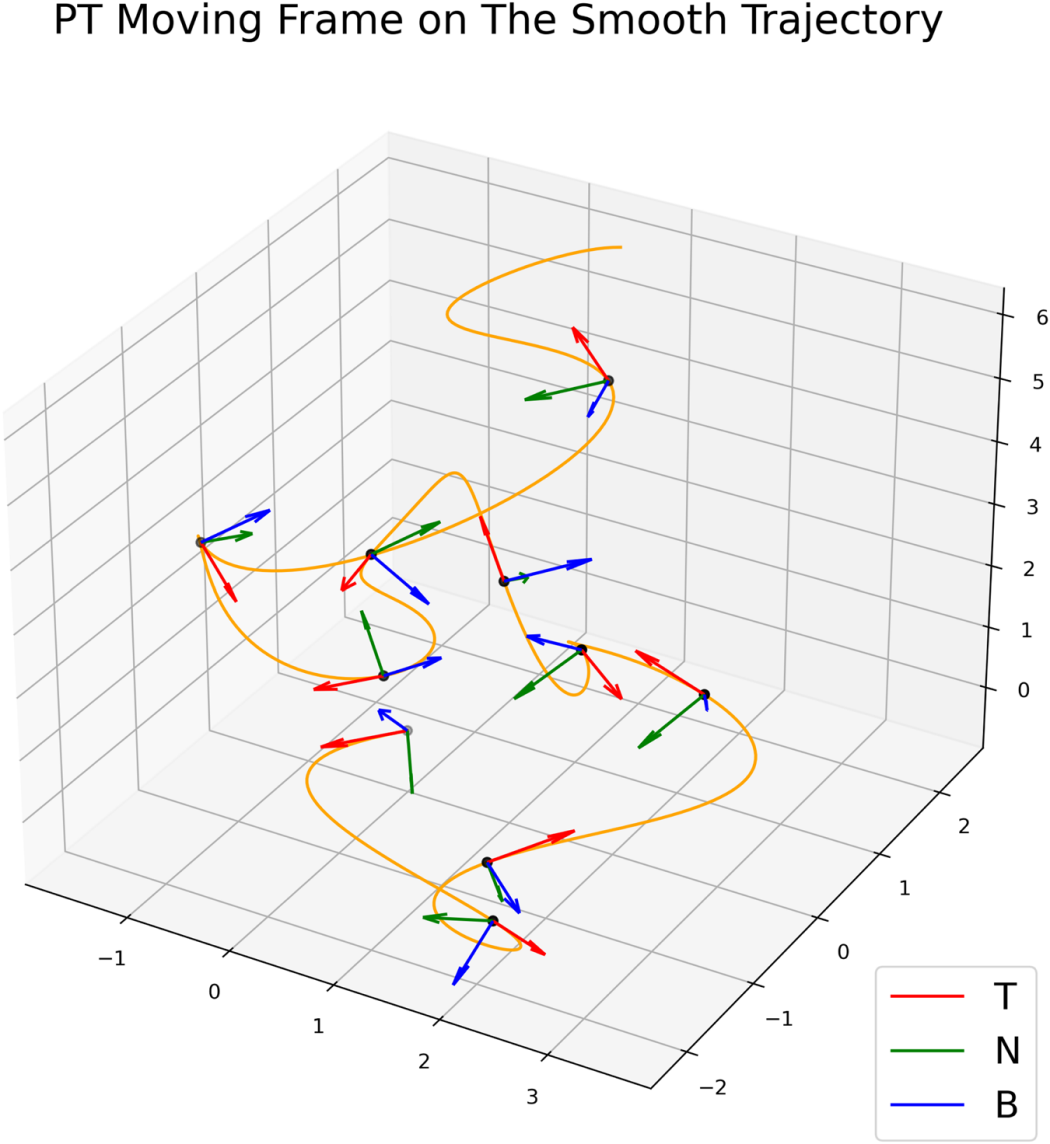
**The construction of PT moving frame on each time point.** The moving frames is moving with the curve and telling us the directions of the object movement.

One of the main ideas in our approach is a frame of reference, which moves along with the curve and tells us the main directions of the movement (Fig. 9). We made this frame of reference using parallel-transport (PT) moving frame algorithm from Hanson and Ma (1995). In brief, we calculated a tangent vector **T_i_** for each point *i* on the curve. Then, set an initial normal vector **N_0_**, which is perpendicular to the first tangent vector **T_0_**. For each sampled point, we calculated the cross product of 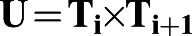. If the length 
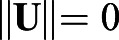
 (i.e. both vectors are parallel) then **N_i+1_**=**N_i_**. Otherwise, to obtain **N_i+1_**, we rotated N_i_ around the vector **U** by the angle *θ*=**T_i_** · **T_i+1_** using the rotation matrix *Rot*(U,*θ*) defined in Hanson and Ma (1995). After we obtained **T_i_** and **N_i_** for each time point *i*, we calculated the vector **B_i_**=**T_i_**×**N_i_** to construct moving frames. The complete algorithm is shown in Algorithm 1.

**Algorithm 1:**
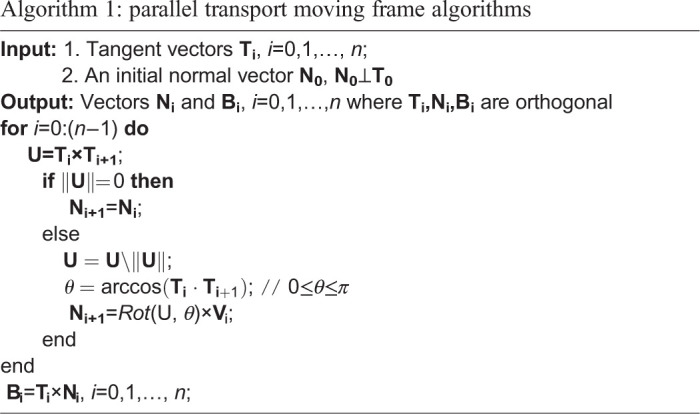
parallel transport moving frame algorithms

#### Orientation normalization

We change the coordinate basis from the standard basis to the PT moving frame basis using simple linear algebra transformation:(1)
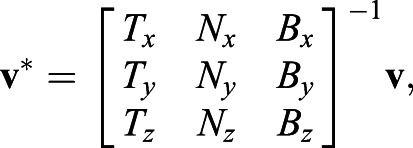
where v* and **v** are the coordinates of the vertex in moving frame and standard basis, respectively. The subscript *x*, *y*, and *z* here are the first, second, and third component of the vectors **T, N**, and **B**.

#### Speed, curvature, and torsion

From the smooth trajectory, we can extract the trajectory features such as speed, curvature, and torsion as defined on Patrikalakis and Maekawa (2009). In summary, speed is the distance traveled per unit of time. Curvature measures the failure of a trajectory to be a straight line, while torsion measures the failure of a trajectory to be planar. Taken together, the curvature and the torsion of a space curve are analogous to the curvature of a plane curve.

### SH decomposition

After orientation normalization, shapes were decomposed by SH transform. To perform SH decomposition, we need to map the object surface to the unit sphere. Before it, we normalize the volume of all objects to one.

#### Spherical parameterization

We used the mean-curvature flow spherical parameterization method from Kazhdan et al. (2012) to maps the object surface to a unit sphere. The result of spherical parameterization is a continuous and uniform mapping between a point on the object surface and a pair of the latitudinal–longitudinal coordinate (*θ*, *φ*) on a unit sphere:(2)
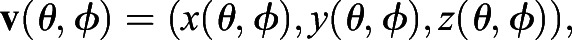
where (*x*(*θ*, *φ*), *y*(*θ*, *φ*), *z*(*θ*, *φ*)) is the Cartesian vertex coordinates.

#### SH expansion

On the unit sphere, each of the *x*(*θ*, *φ*), *y*(*θ*, *φ*), *z*(*θ*, *φ*) can be approximated using the real form SH series:(3)
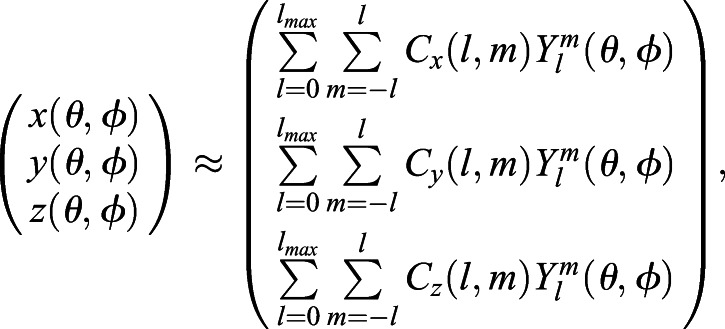


(4)
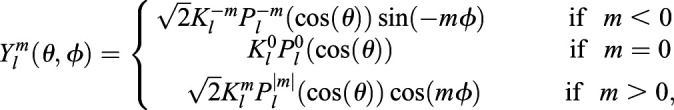

(5)
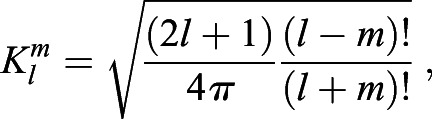
where 
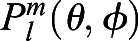
 is the associated Legendre polynomial and *l*_*max*_ is the maximum degree of the SH expansion we want.

The coefficient of degree *l*, order *m*, *C*(*l*, *m*) can be obtained using standard least-square estimation. Using *x*(*θ*, *φ*) as an example, assume that the number of vertices is *n* and *x*_*i*_=*x*(*θ*_*i*_, *φ*_*i*_). We need to find the coefficients 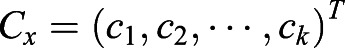, where *c*_*j*_=*C*_*x*_(*l*, *m*). The index *j* for 
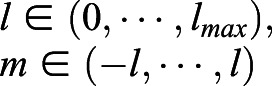
 is obtained from the equation *j*=*l*^2^+*l*+*m*+1. We can obtain the coefficients by solving Eqn 6.(6)
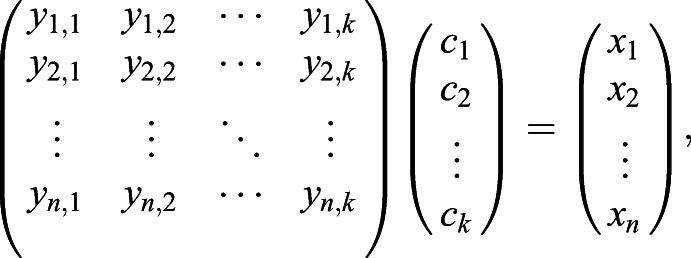
where 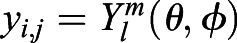, *j*=*l*^2^+*l*+*m*+1, and *k*=(*l*_*max*_+1)^2^. After obtaining the coefficients for each of *C*_*x*_, *C*_*y*_, and *C*_*z*_, we can bundle it into one feature vector of the shape, *C*=(*C*_*x*_, *C*_*y*_, *C*_*z*_).

#### Shape characteristics measures

**Fig. 10.**
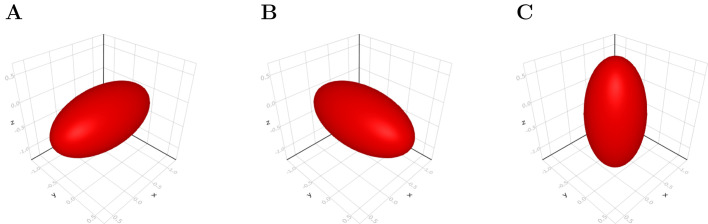
**Shape eccentricity calculated using SH coefficients.** For example, panel A has the eccentricity: *E_xy_*=2; *E_xz_*=2; *E_yz_*=1; (B), *E_xy_*=1=2; *E_xz_*=1; *E_yz_*=2; (C), *E_xy_*=1; *E_xz_*=1=2; *E_yz_*=1=2.

Each coefficient of the expansion retains a shape information corresponding to a particular spatial frequency. The increasing degree of *l* describes the finer scales of shape information. The direction of shape changes can be detected in each of three sets of coefficients, especially *C*_*x*_(1, 1) for deformation in the *x*-direction, *C*_*y*_(1, 0) for *y*-direction, and *C*_*z*_(1,−1) for *z*-direction. If *C*_*x*_(1, 1)=*C*_*y*_(1, 0)=*C*_*z*_(1,−1) and the other coefficients are 0 then the object is a perfect sphere. Based on these three coefficients, we defined three eccentricity index:(7)
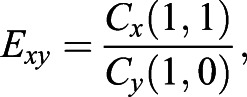
(8)
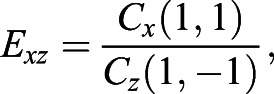
(9)
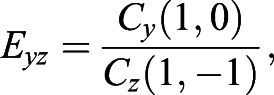
*E*_*xy*_, *E*_*xz*_, and *E*_*yz*_ are the measurements of how much the object deviates from being sphere in *xy*, *xz*, and *yz* plane, respectively (Fig. 10). We can exclude the *E*_*yz*_ because the calculation of *E*_*xy*_ and *E*_*xz*_ already contains all of the eccentricity information.

The difference between shape *i* and *j*, *d*(*i*, *j*), can be calculated using any distance metrics intended for real-valued vector spaces. The most common is the *L*_2_ norm distance:
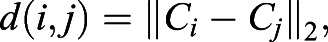
where *C*_*i*_ and *C*_*j*_ are SH coefficients for shape *i* and *j*, respectively. Here, we calculated the rate of shape change, which is defined as *d*(*t*, *t*+1) , where *t*, *t*+1 are two consecutive time points.

#### Statistical analysis

For each cell in the real dataset, we calculated the median, median absolute deviation, twenty-fifth percentile, and seventy-fifth percentile of the speed, curvature, torsion, shape change rate, *E*_*xy*_, and *E*_*xz*_ from all time points. We also counted the number of peaks from the curvature and torsion graph. These values are used as features (26 features in total). Then, we standardized all these features to have mean 0 and standard deviation 1. To show the correlation from each features, we calculated Kendall rank correlation coefficient.

We use UMAP (McInnes et al., 2018) for visualization in a 2D plot. The UMAP parameters that we used: number of neighbors=20, number of components=2, minimum distance=0.1, and the Euclidean distance as metric.

KNN classifier was trained on the shape-movement features on the PT moving frame and on the standard basis to show the performance of our approach. We varied the hyper-parameter *k*=3, 5, 10, 15, 20, 25. A stratified tenfold cross-validation was used to give a set of ten accuracy scores for each hyper-parameter *k*. The accuracy score is the fraction of the correct classifications. From each iteration of cross-validation, we calculated the difference of accuracy scores between these two approaches. The one-sample *t*-test was performed to test whether the accuracy difference was significantly different from 0 or not.

### Datasets

#### Simulated dataset

We created a 3D cell object that moves along a path using open-source software Blender (Community, 2018). First, we created an icosphere object and a path for the object to follow. The speed of the object can be controlled in Blender by adjusting the position curves over time on the Graph Editor menu. Then, we manually protruded the pseudopodia along the object movement. The cell surface texture was produced by adding clouds texture and random noise. We rendered the 3D objects using Eevee render engine in Blender.

The dataset consists of 250 time points and a 3D object at each time point. The 3D objects were saved as triangular mesh objects. In our simulation, the cell moves in the accelerate-decelerate-accelerate-decelerate pattern. The cell starts from a near-spherical shape, protruding a pseudopod when accelerating, and back to near-spherical shape when decelerating (Movies 1 and 2a). The volume of the cell is fixed to be one. We chose the shape object at time point *t*={1, 10, 20, …, 250} as the observed data. The rest of the data were used as the holdout data. Using the simulated dataset, we wanted to verify whether the features extracted from the complete data could be approximated using the features extracted from the smooth trajectory of the observed data. The true trajectory of the cell is defined as the center of the mass of the cell at each of 250 time points. This dataset is available on github (https://github.com/yusri-dh/MovingFrame.jl).

#### Real dataset

The real dataset consists of 3D objects from microscope images of neutrophils. We isolated the neutrophils from the bone marrow of LysM-EGFP mice. Erythrocytes were excluded from harvested bone marrow cells using ACK lysing buffer and density gradient centrifugation (800 ***g*** for 20 min) using 62.5% percoll. The isolated neutrophils were mixed with collagen with 3 × 10^4^ cells/µl. Next, we dropped collagen-cell mixture (10 ul) on the glass bottom dish. After the collagen-cell mixture turned into a gel, we add culture medium to the dish. The dish was incubated at 37°C for 2 h. Before the imaging, the culture medium was replaced with the imaging medium. After the cells were stimulated with GM-CSF 25 ng ml^−1^, LPS 10 µg ml^−1^, or PMA 1 µg ml^−1^, we immediately performed imaging for 90 min at 1-min intervals using two-photon excitation microscope (Nikon A1R MP). We used lens Nikon X20 (Apo LWD 20X/0.95 WI *λ* S), excitation wave length 900 nm, and xy-spatial resolution 0.5 µm. The imaging was performed at 45 µm (15 stacks) in 3 µm steps in the Z-axis direction. Then, we constructed the 3D mesh shape object using a method from Cheng et al. (2020).

We analyzed the cells that were captured in a minimal six consecutive time points (i.e. 233, 398, 293, and 166 cells in saline, GM-CSF, LPS, and PMA group, respectively).

All animal experiments were carried out according to the guidelines approved by the Osaka University Institutional Animal Care and Use Committee.

### Method implementation

The above method was developed as Julia Programming Language package under MIT license (Bezanson et al., 2017). The package can be downloaded using Julia from the Github repository.

We used GaussianProcesses.jl package for the GP smoothing (Fairbrother et al., 2018 preprint). For SH coefficient calculation, we used Julia wrapper for the GNU Scientific Library (GSL) (Galassi, 2009).

For the SE kernel parameter, we set the length-scale=*e*^1.0^ and variance=1.0. We note that the choice of the parameters is not necessarily optimal, but it gives good modeling results in our simulation. For the SH decomposition, we used *l*_*max*_=6.

## Supplementary Material

Supplementary information
