# Supplementary material for: Integrated analysis of cell shape and movement in moving frame
Source: Biol Open. 2021 Mar 26;10(3):bio058512. doi: 10.1242/bio.058512 (PMC8015248; doi:10.1242/bio.058512)
Supplement: Supplementary information [file biolopen-10-058512-s1.pdf]

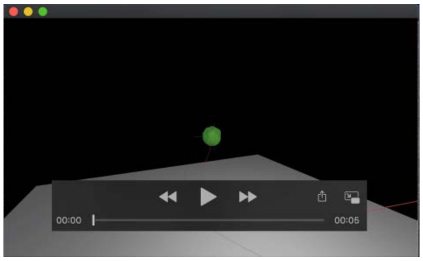

Movie 1. The simulational cell manually created using Blender.

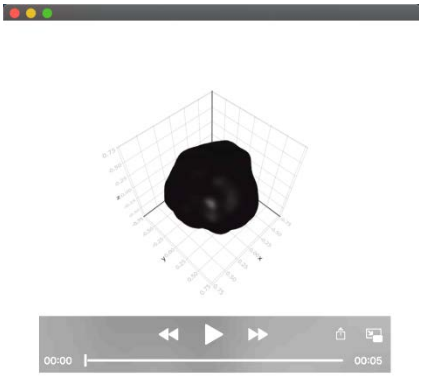

Movie 2a. The simulational cell shape before reorientation. The centers of mass for each timepoints are normalized to origin.

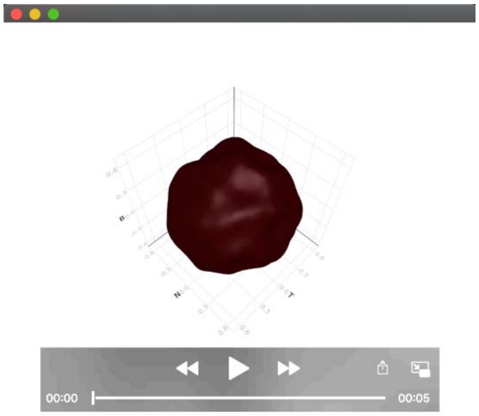

Movie 2b. The simulational cell shape after reorientation. The centers of mass for each timepoints are normalized to origin.

Biology Open • Supplementary information
